# T-Consciousness fields alter germination, growth, and biochemical responses of wheat (*Triticum aestivum* cv. Bahar) under drought stress

**DOI:** 10.1080/15592324.2026.2627034

**Published:** 2026-02-09

**Authors:** Sara Torabi, Mohammad Ali Taheri, Farid Semsarha, Aidin Hamidi, Mubshar Hussain, MirSaeid Moghadampour, Fariba Mohammadifard

**Affiliations:** a Department of Plant Biology, School of Biology, College of Sciences, University of Tehran, Tehran, Iran; b Sciencefact R&D Department, Cosmointel Inc. Research Center, Toronto, Ontario, Canada; c Institute of Biochemistry and Biophysics (IBB), University of Tehran, Tehran, Iran; d Agricultural Research, Education and Extension Organization (AREEO), Seed and Plant Certification and Registration Institute (SPCRI), Karaj, Iran; e Department of Agronomy, University of Agriculture, Faisalabad, Pakistan; f Islamic Azad university, Rasht Branch, Rasht, Iran; g Department of Horticultural Science, Faculty of Plant Production, Gorgan University of Agricultural Sciences and Natural Resources, Gorgan, Iran

**Keywords:** T-Consciousness field, drought stress, wheat, growth, superoxide dismutase, pigment

## Abstract

The existence of consciousness or mind-like properties in plants remains a debated topic in plant biology. This study examined a hypothesis involving nonfrequency T-Consciousness Fields, proposing that information transmitted through these fields may influence plant responses. Using the Faradarmani Consciousness Field (T1) and the T-Consciousness Charge Field (T2), two experiments were conducted in a completely randomized design to assess their effects on wheat (*Triticum aestivum* cv. Bahar) under drought stress. The germination test was carried out in March, and the subsequent pot experiment was conducted in September 2025 in Gorgan and Guilan Provinces, Iran. In the first experiment, seeds were exposed to PEG-induced drought stress (0, −0.6, and −1.2  MPa) for 8 d, with or without T1 and T2, to evaluate germination and early growth. In the second experiment, seedlings grown in pots were subjected to three weeks of drought by withholding irrigation, with untreated plants serving as controls. Growth parameters, chlorophyll, carotenoid, total protein, and superoxide dismutase (SOD) activities were measured. The results obtained were processed statistically via one-way ANOVA. Severe drought reduced final and mean daily germination by about 40%, whereas T2 significantly improved both (*p* < 0.05). At −0.6 MPa, shoot and root lengths increased by approximately 70% and 46%, respectively, with significant greater enhancement under T2 (*p* < 0.05), whereas effects under more severe stress were limited. Under nonstress conditions, T2 markedly increased seedling growth and vigor, with 2–3-fold increases in root and shoot dry weights and 3–4-fold increases in seedling vigor indices compared with those of the control. In the pot experiments, T2 increased shoot length by ~25% and chlorophyll and carotenoid contents by ~60%, while T1 increased protein content by ~25%. Both fields elevated SOD-specific activity by ~50%. Overall, T1 and T2 improved germination, growth, and biochemical traits, indicating their potential to mitigate drought stress in wheat; thus, their application could be recommended as a qualitative strategy to enhance wheat performance under water-limited conditions.

## Introduction

Consciousness is one of the most complex concepts discussed in science. Although many efforts have been made to understand its nature and how it emerges, no unified definition has yet been proposed.^1^ In fact, since the 16th and 17th centuries, extensive debates have taken place regarding the nature and function of consciousness.^2^ While the local view in neuroscience focuses on the brain to determine how consciousness arises from neural activity, the nonlocal view considers consciousness a phenomenon that extends beyond the brain and may permeate the very fabric of the cosmos.^3^ According to Taheri's perspective, consciousness does not arise from neural activity; rather, it is a fundamental element of the universe from which matter, energy, and information originate. In this approach, in addition to the physical part of the universe—comprising particles and waves, which have a frequency-based nature—there exists another nonfrequency-based aspect, such as information, mind, consciousness, etc.^4^

For example, to better understand this, one can consider a computer that has both hardware and software components. Although the software has no physical or directly observable form, the functioning and operation of the computer depend on it. Similarly, observable behaviors in the interactions of inanimate matter, as well as at the level of living organisms, indicate a precise software-like program with accurate information processing. This software-like component that processes information is considered the mind in Taheri's view. In other words, every part—from inanimate particles and waves to living beings—requires this software-like component to perform specific functions.^5^ Indeed, there are various levels of consciousness and mind, which, in nonhuman animals and plants, operate at the level of instinct.

It should be noted that the most significant challenge in consciousness studies is the possibility of practical and experimental testing. As mentioned, many of the phenomena examined in science are objective and third-person in nature, whereas consciousness is described as a first-person experience.^6^ Taheri's theory of Consciousness Fields provides an opportunity for practical and experimental assessments of consciousness. In this view, diverse T-Consciousness Fields (TCFs) exist with various functions and are considered subsets of the Cosmic Consciousness Network. The effects of these fields are initiated through the mind of the announcer, referred to as the *Faradarmangar* (a trained individual). It is hypothesized that the information transmitted by these fields is received by the subject's mind under study, which may subsequently lead to observable changes in the samples. Experimental samples can include biological systems, living organisms, and even matter and waves.^7^^,^^8^ This characteristic has encouraged researchers to design experiments aimed at empirically investigating this theory.

Drought stress is one of the major abiotic stresses affecting crop production worldwide. According to the Intergovernmental Panel on Climate Change, climate warming has altered precipitation patterns, resulting in persistent droughts across many regions. Moreover, approximately half of the global population experiences severe water scarcity for part of each year due to the combined influence of climatic and nonclimatic factors.^9^ Wheat is one of the most important cereal crops, providing a major source of food and nutrition for people worldwide.^10^ In 2020, it was estimated that nearly one-fifth of global farms cultivated with wheat; however, this number is projected to decline by about 4% by 2030, decreasing from approximately 135 million to 130 million farms.^11^ Meanwhile, with increasing global population, it is estimated that wheat yield must increase from the current 3.3 tons per hectare to about 5 tons per hectare by 2050 to meet food demand.^12^

Water deficit has detrimental effects on all developmental stages of wheat, from germination to maturity.^13^^,^^14^ Such conditions reduce nutrient availability in the root zone and disrupt translocation through the xylem and phloem, thereby impairing nutrient metabolism in plant cells and tissues.^15^ Numerous attempts have been made to reduce the adverse effects of drought stress on cereal crops. For instance, a recent study showed that although deficit irrigation limits wheat physiological performance, nutrient uptake, and grain quality, these effects can be effectively mitigated through integrated nutrient management, which enhances photosynthesis, stress tolerance, and water use efficiency under drought conditions.^16^^,^^17^

Seed germination proceeds through a series of interconnected processes, including imbibition, metabolic activation, and intraseminal growth, ultimately culminating in radicle protrusion. Water uptake is a fundamental prerequisite for the initiation and completion of germination. This initial phase is critical for creating favorable conditions for metabolic activity and biochemical reactions and for stabilizing the conformation of macromolecules.^18^^,^^19^ Germination and early seedling growth are highly sensitive stages of plant development, highlighting the importance of drought tolerance during early growth.^20^ Water deficiency at the time of sowing adversely affects seed germination and alters physiological and biochemical processes during seedling establishment, ultimately leading to reduced growth, impaired development, and lower overall productivity.^21^ Given the importance of this growth stage, numerous studies have examined the detrimental effects of drought stress on seed germination in various agricultural crops, such as maize (*Zea mays* L.), sunflower (*Helianthus annuus* L.), wheat (*Triticum aestivum* L.), etc.^22-24^ Achieving a higher yield in agriculture greatly depends on rapid seed germination; therefore, strategies such as seed priming or pretreatment with nanoparticles have been proposed to increase germination rates.^25^

Additionally, various metabolic processes, such as photosynthesis, are disrupted under these conditions, and increased production and accumulation of reactive oxygen species cause oxidative damage.^26^ Although plants are unable to move or escape under environmental stress conditions, they possess various mechanisms that enable them to defend themselves and increase their survival. These responses occur at multiple levels, from cells and organs to the whole plant, such as stomatal regulation, emission of volatile organic compounds, and the production of molecular signals by the roots.^27^ Under abiotic stresses, the balance between the production and scavenging of reactive oxygen species (ROS) is disrupted. One of the ways to maintain this balance is through the antioxidant defense system, which includes enzymatic antioxidants such as superoxide dismutase (SOD), catalase (CAT), and ascorbate peroxidase (APX), as well as nonenzymatic antioxidants such as glutathione and ascorbate.^28^

The effects of drought stress on wheat germination and growth have been reported in several studies.^29^^,^^30^ The novel aspect of this study is the investigation of the effects of nonfrequency TCFs on wheat plants under drought stress. For this purpose, two separate experiments were conducted. In the first stage, drought stress was induced using polyethylene glycol (PEG), which reduces water availability by creating osmotic stress.^31^ The effects of the TCFs on the germination and seedling growth of wheat were evaluated over an 8-d period under both drought stress and normal conditions (receiving distilled water). After the initial changes were observed, a second experiment was designed to assess the effects of these fields under real drought conditions. In this stage, the growth and biochemical indices of seedlings exposed to drought by withholding irrigation for three weeks were measured, both with and without the influence of the TCFs. The empirical evidence from these experiments primarily evaluates the potential of these fields to alleviate the detrimental effects of drought stress and, second, may indicate the possibility of information reception by plants under the influence of T-Consciousness Fields.

## Materials and methods

### Application of T-consciousness Fields

Two types of T-Consciousness Fields, including Faradarmani (T1) and T-Consciousness Charge (T2), were applied to the treated samples according to the COSMOintel Research Center's protocol.1.www.cosmointel.com.^32^ The effects of both fields begin with brief and instantaneous attention to the subject under study. For T2 application, a substance must first be exposed to this field, and subsequent application of this substance may alter the behavior of the treated samples. In this experiment, owing to the experimental design and the use of plants, water was used as the medium to convey the information provided by the T2 treatment. For the germination experiment, the water was charged at the start of the experiment, whereas in the pot experiment, the water was exposed to the T-Consciousness Charging Field approximately one week prior to the beginning of the experiment. After this brief and instantaneous charging process, the water was labeled as T2, stored under normal laboratory conditions, and handled in the same manner as the control water. No substances, additives, or physical interventions were introduced into the water, and all experimental conditions (storage containers, temperature, light exposure, and handling procedures) were kept identical between the control and treated samples. The field treatments were initiated at the start of the experimental period and maintained continuously until completion. Being neither matter nor energy, these fields can exert their influence regardless of distance. It should be noted that access to these treatments is freely provided by this research center to facilitate experimental reproducibility. In this experiment, the person applying the TCFs was not present in the laboratory, and the expert who recorded the data labeled only the experimental groups as C, T1, and T2 without any knowledge of the theory behind TCFs, which helped reduce the potential for bias in data recording. The treatment codes (abbreviations) were not revealed until all the data had been fully recorded and finalized. It should be noted that English language refinement was performed using ChatGPT (OpenAI GPT-5.1). No scientific interpretation or content generation was delegated to the tool.

### Germination experiment design

Seeds of the bread wheat cultivar Bahar certified class were obtained from Pishro Kesht Alborz Company, Karaj, Iran. This cultivar is suitable for temperate regions, is susceptible to yellow rust disease, and is classified as having moderate drought tolerance. This experiment was conducted in March 2025 at Gorgan University of Agricultural Sciences and Natural Resources, Gorgan, Iran. The seeds were surface-sterilized with 70% ethanol for 2 min and rinsed twice with autoclaved distilled water. The experiment was conducted in a completely randomized design with three replicates for each experimental group. Nine experimental groups included: 1) control (0 MPa), 2) control with T1 treatment, 3) control with T2 treatment, 4) −0.6 MPa drought stress, 5) −0.6 MPa drought stress with T1 treatment, 6) −0.6 MPa drought stress with T2 treatment, 7) −1.2 MPa drought stress, 8) −1.2 MPa drought stress with T1 treatment, and 9) −1.2 MPa drought stress with T2 treatment.

The standard seed germination test was conducted in 9-cm Petri dishes according to the International Seed Testing Association^33^ rules, in which 25 seeds were placed on two layers of filter paper in each Petri dish.^34^ The drought stress groups received 10 mL of polyethylene glycol (PEG) solution^35^^,^^36^ at two levels, with or without TCF treatment, while the two control groups received 10 mL of autoclaved distilled water instead of the PEG solution. The Petri dish lids were sealed with Parafilm to prevent evaporation and were placed in a germinator under 16/8 h light/dark conditions at 20 °C. To maintain moisture and prevent the accumulation of PEG effects, the filter papers were replaced every other day, and 10 mL of the respective PEG solution or 10 mL of distilled water was added to the experimental groups.^34^^,^^37^ Germinated seeds were counted daily at a fixed time for 8 d. Seeds were considered germinated when the radicle reached a length of 1 mm. The final germination percentage (FGP) and mean daily germination (MDG), which are indices of the daily germination rate, were calculated as follows^38^:

FGP=Ng/Nt×100,

where Ng​ is the number of germinated seeds and Nt is the total number of seeds.

MDG=FGP/D,

where FGP is the final germination percentage and *D* is the number of days until the end of the germination test period.

At the end of the period, the lengths of the shoots and roots were measured in each replicate using a caliper with 0.1 mm accuracy. In addition, weights were recorded using a digital balance with a precision of 0.001 g. The shoots and roots from each replicate were placed separately on foil and dried at 75 °C for 24 h, and the biomass of the shoots and roots was reported.^21^ The seedling length vigor index (SLV) and seedling weight vigor index (SWV) were calculated using the following formulas^39^:

SLV:seedlinglength×FGP,

SWV:seedlingdryweight×FGP.

### Pot experiment design

The experiment was conducted in September 2025 at the University of Guilan, Guilan, Iran. The seeds were sterilized with 20% sodium hypochlorite and rinsed with distilled water. Plastic pots with a diameter of 13.5 cm and a height of 12.5 cm were selected, and five seeds were sown in each pot containing a mixture of sand and peat (2400 g) at a ratio of 2:1. The pots were maintained under greenhouse conditions with a daytime temperature of 25 °C, a nighttime temperature of 18 °C, a humidity of 70%, and a photoperiod of 16 h light and 8 h darkness. The experimental groups included the control (without Fields treatment), T-Consciousness Charge Field, and Faradarmani Consciousness Field, each with five pot replicates containing five seeds. As mentioned in the section on the application of T-Consciousness Fields, to apply these fields, it is sufficient to briefly and intentionally specify which samples are influenced by the TCFs. In the case of the T-Consciousness Charge Field, the water used for this experimental group is exposed to the field and thus becomes “charged.” It is then labeled and separated from the control sample (which is not exposed to the field). This type of charging has a consciousness-based, nonphysical nature, which, according to the theory, conveys certain information to the samples, consequently altering the properties of the water. With respect to the Faradarmani Consciousness Field, it is only necessary to designate the experimental pots intended for exposure. In other words, the influence of these nonfrequency fields is initiated through the mind by brief and momentary attention lasting only a few seconds. The pots were irrigated every other day to 100% field capacity, as determined by weighing, which was approximately 800 mL. As the experiment focused on approximately short-term seedling responses to drought stress, no additional fertilizers were applied.^40^ After three weeks, drought stress was imposed by withholding irrigation, a method commonly employed in greenhouse experiments to assess seedling responses to water deficit.^41-43^ The stress treatment continued for three weeks. At the end of the period, six-week-old seedlings were harvested. One pot in the control group contained no seedlings and was excluded from further analyses. At least three seedlings from each experimental group were frozen in liquid nitrogen for biochemical analyses and transferred to the Department of Agriculture, Medicinal Plants and Drugs Research Institute, Shahid Beheshti University, Tehran, Iran, where the biochemical indices were measured. The samples were stored at −80 °C for further experiments. Sampling was performed using uniform seedlings, and only plants with fully expanded leaves were selected in order to minimize variability. The aboveground parts, including shoots and leaves, were separated from the roots, and their lengths were measured using a caliper with 0.1 mm precision. For dry weight measurement, the separated parts were placed on foil and dried in an oven at 70 °C for 24 h. Dry weight was determined using a digital balance with a precision of one thousand per gram, and the results were reported per plant.

#### Chlorophyll and carotenoid content measurement

For pigment extraction, 0.5 g of fresh leaf tissue was thoroughly ground in a porcelain mortar with liquid nitrogen until a uniform powder was obtained.^44^ Subzero grinding minimized pigment degradation during sample preparation.^45^ The resulting powder was transferred into a Falcon tube, and 20 mL of 80% acetone was added for extraction.^46^ The samples were then centrifuged at 3000 rpm for 5 min to separate the liquid phase. The absorbance of the extracted solution was measured at wavelengths of 663, 645, and 470 nm using an ELISA reader. Finally, the contents of chlorophyll *a*, chlorophyll *b*, total chlorophyll, and carotenoids were calculated according to the following equations and expressed as µg mL^−1^ of extract.^47^

Chla(µg/mL)=(12.25×A663)−(2.79×A645),

Chlb(µg/mL)=(21.21×A645)−(5×A663),

Chltotal(µg/mL)=Chla+Chlb,

Carotenoid(µg/mL)=((1000×A470)−(1.8×Chla)−(85.02×Chlb))/198.

### Extract preparation

Enzymatic and protein extracts were prepared following the method of Gajewska et al.^48^ with slight modifications. Half a gram of fresh leaf tissue was thoroughly ground in a porcelain mortar with liquid nitrogen. Then, 2 mL of 50 mM potassium phosphate buffer (pH = 7.5) containing 2 mM EDTA and 1% (w/v) polyvinylpyrrolidone (PVP) was added. The homogenized mixture was centrifuged at 13,000 rpm for 10 min at 4 °C. The supernatant was collected and stored at –80 °C for subsequent determination of protein content and antioxidant enzyme activity.

### Total protein measurement

The protein content of the enzymatic extracts was determined using the Bradford method.^49^ This method is based on the colorimetric reaction of Coomassie Brilliant Blue G250 with amino acids in proteins, resulting in a blue-colored complex and a shift in absorbance at 595 nm. To prepare the Bradford reagent, 10 mg of Coomassie Brilliant Blue G250 was dissolved in 5 mL of 96% ethanol. Then, 30 mL of distilled water and 200 mg of sodium hydroxide were added, and the solution was completely dissolved. Next, 10 mL of phosphoric acid was added dropwise, and finally, the volume was adjusted to 500 mL with distilled water. The prepared solution was stored at 4 °C in the dark. For protein measurement, 250 μL of Bradford reagent and 25 μL of the enzymatic–protein extract were added to each well of a 96-well plate in three replicates. After 3 min, the absorbance at 595 nm was measured using an ELISA reader. A standard curve was prepared using bovine serum albumin (BSA) at concentrations of 0, 2.5, 5, 7.5, 10, 12.5, 15, 17.5, and 20 μg/mL. For each concentration, 250 μL of Bradford reagent was added to the well, followed by the addition of 25 μL of the standard solution in triplicate. After reading the absorbance at 595 nm, the standard curve was plotted. The protein content of the samples was calculated using the standard curve and expressed as milligrams of protein per gram of fresh tissue (mg protein g⁻¹ FW).

### Superoxide dismutase (SOD) activity assay

The activity of superoxide dismutase (SOD) was measured according to the method of Kono^50^ with slight modifications. The riboflavin/nitro blue tetrazolium (B2/NBT) method for assaying SOD activity is a simple and practical approach, especially for crude extracts.^51^ Superoxide anion radicals generated from light-excited riboflavin are used to study the effect of luminescence on the photoreaction of nitro blue tetrazolium (NBT). NBT acts as a scavenger indicator and is reduced by superoxide radicals. Its reduction leads to an increase in absorbance at 560 nm, a process that can be inhibited by SOD.^52^ Fifty microliters of enzymatic extract were mixed with 1 mL of a reaction mixture containing 50 mM phosphate buffer (pH 7.8), 12 mM L-methionine, 750 μM nitro blue tetrazolium chloride (NBT), and 1 mM EDTA. Then, 10 μL of 2 mM riboflavin was added (all the chemicals were purchased from Sigma-Aldrich). The mixture was transferred to a glass cuvette and exposed to fluorescent light for 15 min. After incubation, the absorbance was measured at 560 nm using a spectrophotometer. SOD activity was defined as one unit of SOD that induces 50% inhibition of the B2/NBT system^53^ The specific activity of the enzyme was expressed as SOD units per milligram of protein. To calculate the SOD content, the inhibition rate of different concentrations of the enzymatic extract was first determined using the following formula:

$$\%\;\mathrm{Inhibition}\;(A)=\;\frac{\mathrm{Ac}-\mathrm{Ax}}{\mathrm{Ac}}\times 100,$$

where Ac​ is the absorbance of the reaction mixture without the enzymatic extract and Ax is the absorbance of the reaction mixture containing x μL of the enzymatic extract. The final specific activity of SOD was calculated as follows:

B=A/C,

where *B* is the specific activity of the enzyme expressed as a unit of SOD per mg of protein, and *C* is the protein concentration in mg/mL.

### Statistical analysis

The data were analyzed using GraphPad Prism version 10.4.1. The results are presented as mean ± standard deviation (SD) of at least three replicates. In the germination experiment, each experimental group included three Petri dishes, each containing 25 seeds, while in the pot experiment, each group included five pots, each containing five seeds. Differences between treatment means were evaluated using one-way analysis of variance (ANOVA) followed by Tukey's multiple comparison test. Differences were considered statistically significant at *p* < 0.05.

## Results

As shown in Table 1, in the germination test, the first level of drought stress (–0.6 MPa) did not have a significant effect on the final germination percentage. However, the second level (–1.2 MPa) significantly reduced final germination (*p* < 0.05). Under this level of stress, the TCFs improved germination, and the effect of the T-Consciousness Charge Field (S2T2) was statistically significant compared to the stressed condition without the fields (S2) (*p* < 0.05). Similar trends were observed in the calculation of the mean daily germination.

**Table 1.** Effect of drought stress and T-Consciousness Fields on final germination percentage (FGP), mean daily germination (MDG), and growth indices in wheat.

Characters/treatments	C	CT1	CT2	S1	S1T1	S1T2	S2	S2T1	S2T2
FGP	66.7 ± 8.3 a	68.0 ± 5.7 ab	80.0 ± 4.0 a	80.0 ± 11.3 a	78.0 ± 2.8 a	78.7 ± 4.6 a	40.0 ± 0.0 c	46.0 ± 2.8 bc	72.0 ± 5.7 a
MDG	8.7 ± 1.2 a	8.5 ± 0.7 ab	10.3 ± 0.6 a	10 ± 1.4 a	10 ± 0 a	10.3 ± 0.6 a	5 ± 0 c	6 ± 0 bc	9.5 ± 0.7 a
Root length (mm)	23.3 ± 4.2 d	23 ± 4.2 d	78.3 ± 38.9 a	34 ± 19.8 c	28.5 ± 7.8 cd	48 ± 4 b	22.5 ± 2.1 d	21 ± 0.0 de	14 ± 4.2 e
Shoot length (mm)	32.3 ± 0.6 d	52 ± 14.1 c	118.3 ± 11.1 a	55 ± 8.5 c	48.5 ± 14.8 c	79 ± 11.5 b	27.5 ± 0.7 de	25.5 ± 0.7 de	23 ± 5.7 e
Root dry weight (g)	0.12 ± 0.015 ab	0.17 ± 0.061 ab	0.27 ± 0.066 a	0.18 ± 0.083 ab	0.17 ± 0.082 ab	0.22 ± 0.063 ab	0.1 ± 0.025 ab	0.094 ± 0.0083 b	0.17 ± 0.088 ab
Shoot dry weight (g)	0.11 ± 0.019 bc	0.25 ± 0.11 ac	0.39 ± 0.089 a	0.25 ± 0.1 ac	0.26 ± 0.073 ac	0.3 ± 0.037 ab	0.12 ± 0.015 bc	0.093 ± 0.009 c	0.17 ± 0.08 bc
Seedling weight vigor index	0.91 ± 0.14 b	1.3 ± 0.41 b	2.7 ± 0.62 a	1.3 ± 0.37 b	1.4 ± 0.29 ab	2.1 ± 0.38 ab	0.83 ± 0.14 b	0.78 ± 0.008 b	1.7 ± 0.54 ab
Seedling length vigor index	3705.3 ± 780 c	5141 ± 1658 bc	15744.7 ± 3230 a	7279 ± 3258 bc	5977 ± 1608 bc	9989.7 ± 75.6 b	1990 ± 110 c	2155 ± 123 c	2614 ± 514.8 c

Means sharing the same letter are not significantly different according to Tukey's multiple comparison at *p* ≤ 0.05. Here, C = (control; 0 MPa), CT1 = Control with Faradarmani (T1), CT2 = control with T-Consciousness Charge Field (CT2), S1 = –0.6 MPa, S1T1 = –0.6 MPa with T1, S1T2 = –0.6 MPa with T2, S2 = –1.2 MPa, S2T1 = –1.2 MPa with T1, and S2T2 = –1.2 MPa with T2.

Regarding growth traits, as observed, the first level of drought stress significantly increased root and shoot lengths compared to the control (0 MPa). This length increase was further enhanced under T2 (*p* < 0.05). Under the control conditions, T1 had no effect on root length but significantly increased shoot length (*p* < 0.05). Under the same conditions, T2 significantly increased both root and shoot lengths (*p* < 0.05). Conversely, at the higher drought stress level, a decreasing trend in root and shoot lengths was observed under the influence of these fields.

Similarly, with respect to dry weight, the control under T2 (CT2) was significantly higher than that of the condition without fields (C) (*p* < 0.05). This increase in seedling weight was also observed at both stress levels, although the changes were not statistically significant. The calculation of the seedling vigor length index showed that under the control (CT2) and first stress level conditions (S1T2), this index was significantly higher under the T-Consciousness Charge Field compared to control without fields (C). The highest seedling vigor weight index was observed in the control under T2 (CT2) (*p* < 0.05). Under stress conditions, these indices showed an increasing trend with consciousness fields, but the differences were not statistically significant.

**Figure 1.**
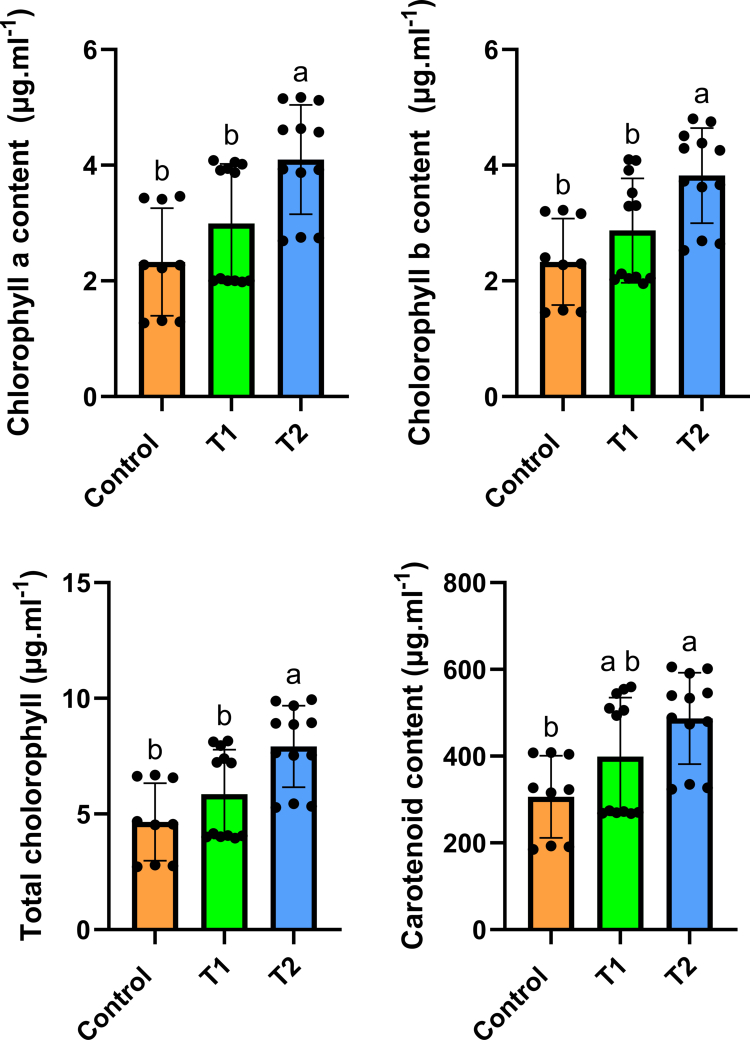
Effects of Faradarmani Consciousness Field (T1) and T-Consciousness Charge Field (T2) on chlorophyll *a*, chlorophyll *b*, total chlorophyll, and carotenoid under drought stress. Means sharing the same letter are not significantly different at the 5% level.

Table 2 shows the growth indices measured in the pot experiment. Similar to the germination test, although changes in dry weight under the influence of TCFs were not significant under drought stress, a significant increase in shoot length was observed under the T-Consciousness Charge Field (*p* < 0.05). Figure 1 illustrates the changes in chlorophyll and carotenoid contents. As shown, in the samples subjected to T2, the chlorophyll *a*, chlorophyll *b*, total chlorophyll, and carotenoid contents were significantly higher than those in the control samples and the samples without field treatment (*p* < 0.05). This increase was also observed under the Faradarmani treatment (T1), but the changes were not statistically significant.

**Table 2.** Growth traits in the experimental groups of the pot study under drought stress.

Characters/treatments	Control	T1	T2
Root length (cm)	19 ± 10 a	18 ± 7.1 a	18 ± 4.9 a
Shoot length (cm)	21.8 ± 4.5 b	19.4 ± 4.25 b	27.3 ± 3.88 a
Root dry weight (mg/plant)	44 ± 29 a	32 ± 15 a	31 ± 19 a
Shoot dry weight (mg/plant)	76 ± 32 a	63 ± 21 a	86 ± 33 a

Control = drought without the influence of T-Consciousness Fields, T1 = drought with the Faradarmani Consciousness Field, and T2 = drought with T-Consciousness Charge Field. Means sharing the same letter are not significantly different at the 5% level.

**Figure 2.**
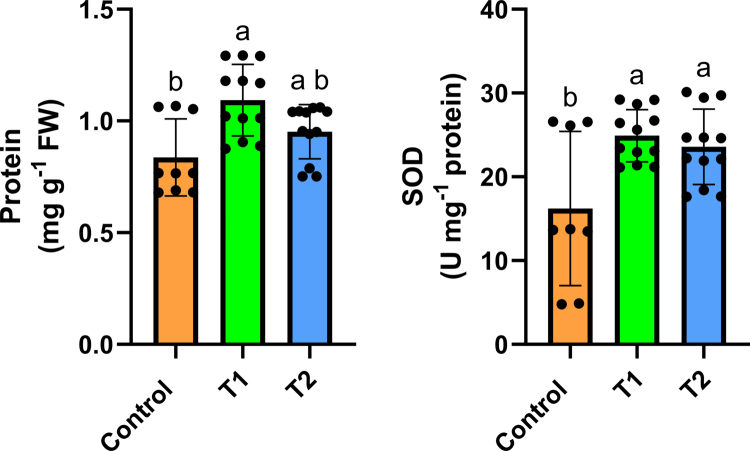
Changes in protein content and superoxide dismutase (SOD) activity under the effects of Faradarmani Consciousness Field (T1), T-Consciousness Charge Field (T2), and without field treatment (control). Means sharing the same letter are not significantly different at the 5% level.

Figure 2 shows the protein content and antioxidant enzyme superoxide dismutase (SOD) activity under drought stress in the control and treatment groups exposed to Faradarmani Consciousness Field (T1) and T-Consciousness Charge Field (T2). Table 3 provides a detailed overview of superoxide dismutase (SOD) activity parameters in the control and in samples treated with TCFs. These fields increased protein content compared to the control, and this enhancement was statistically significant for T1 (*p* < 0.05). Similarly, both fields caused a significant increase in SOD enzyme activity (*p* < 0.05).

**Table 3.** Changes in superoxide dismutase (SOD) activity parameters in control and experimental treatments.

Sample	% Inhibition	Protein (mg ml^−1^)	SOD activity (unit ml^−1^)	SOD specific activity (unit mg^−1^)
Control	37.31 ± 27.74	0.85 ± 0.18	14.93 ± 11.10	16.22 ± 9.19
T1	64.41 ± 4.38	1.06 ± 0.19	25.76 ± 1.75	24.91 ± 3.12
T2	57.27 ± 16.92	0.95 ± 0.12	22.91 ± 6.77	23.60 ± 4.50

Control = drought without the influence of T-Consciousness Fields, T1 = drought with Faradarmani Consciousness Field, and T2 = drought with T-Consciousness Charge Field.

## Discussion

This study investigated the effects of nonfrequency T-Consciousness Fields on wheat plants under drought stress. According to the obtained results, a moderate level of stress did not have a detrimental effect on seed germination, whereas the second, more severe level of stress caused a significant reduction in germination compared to the 0 MPa condition. This finding is consistent with previous studies reporting that reduced water potential adversely affects wheat germination.^54^^,^^55^ A wide range of physical and chemical interventions have been employed to investigate changes in seed germination and seedling growth parameters. For instance, cold argon plasma has been reported to increase the final germination percentage of soybean cultivars.^56^ Moreover, electromagnetic field exposure has been shown to accelerate germination and increase biomass accumulation in wheat seedlings,^57^ and ultrasound treatment has been demonstrated to increase drought tolerance in wheat seedlings.^58^ To our knowledge, this is the first study to evaluate the effects of nonfrequency-based fields on seed germination. The T-Consciousness Fields alleviated the detrimental effects of severe drought stress on germination, leading to higher mean daily germination and final germination percentages in the treated samples, with a statistically significant improvement observed for the Charge Field. It is well established that there is a direct relationship between germination percentage, proper early growth stages, and successful establishment of plants in the field.^59^ The observed increase in shoot and root lengths under control (0 MPa) and mild stress conditions is consistent with another study in which 7-d-old wheat seedlings exposed to the Faradarmani Consciousness Field exhibited greater length.^60^

A comparison between the two PEG levels showed that shoot and root growth increased under –0.6 MPa conditions, which agrees with studies reporting that moderate PEG levels can enhance shoot and root growth.^37^ Ahmed et al.^61^ reported that although polyethylene glycol–induced osmotic stress generally reduces seedling growth in bread wheat, some genotypes maintain or exhibit increased root length, indicating an adaptive response to enhance water acquisition under low water potential. Furthermore, researchers have found that different wheat lines exhibit varying responses to PEG treatment^62^; some genotypes, when exposed to 5% or 10% PEG concentrations, have maintained or even improved their germination rates under these drought stress–simulating conditions.^63^ Moreover, other studies have shown that PEG pretreatment can increase the physiological traits of seedlings and improve germination rates.^64^^,^^65^ This suggests that PEG treatment may increase seedling tolerance to stress, making it a useful tool for improving germination under challenging environmental conditions such as drought.^37^

Seedling vigor indices have been widely measured to evaluate the effectiveness of treatments, such as seed priming, in enhancing germination rates and early seedling growth traits in wheat under stress conditions.^66^ The increase in seedling vigor length and weight indices under nonstress conditions also suggests that these fields can promote growth and biomass production. It is worth mentioning that although PEG solutions reduce water potential and can simulate dehydration or osmotic stress, but they do not replicate the full complexity of soil drought.^67^ To further investigate the potential of these fields in improving drought tolerance, a second, longer-term experiment was designed using pot design, in which biochemical parameters were also evaluated.

The growth traits of the seedlings under drought stress in the pot experiment were consistent with those in the germination test, showing an increase in shoot length in the treated samples. In general, shoot growth is reduced under drought and osmotic stress conditions due to impaired water uptake, restricted cell expansion, and metabolic limitations.^68^ In particular, a growing body of evidence indicates that drought and osmotic stress exert significant adverse effects on wheat shoot elongation across developmental stages and genotypes.^21^^,^^69^ Access to ATP is particularly critical during germination and early growth stages.^70^ Since the photosynthetic system is not yet fully developed at these stages, the energy supply relies mainly on mitochondrial respiration.^71^ Under drought stress, in addition to reduced water absorption and delayed metabolic activity, the production of reactive oxygen species (ROS) exerts damaging effects on mitochondria.^72^ Consequently, energy limitation can reduce the growth rate.^73^ Considering the improvement in growth indices, it is possible that the samples exposed to T-Consciousness Fields experienced less energy deficiency. Previous studies on the HEK-293 cell line have shown that TCFs increased ATP production and inhibited apoptosis under microgravity stress conditions.^74^

The contents of photosynthetic pigments, namely, chlorophylls, were higher in plants exposed to the T-Consciousness Fields. Under water deficit conditions, stomatal closure is a well-known adaptive mechanism that leads to reduced photosynthetic activity.^75^ Drought stress has been reported to affect photosynthetic pigments by inducing oxidative stress, which damages the chloroplast membrane, photosynthetic proteins, and pigments.^76^ To mitigate these adverse effects, researchers have explored several strategies ranging from nutrient modulation to nanomaterial.^77^^,^^78^ Carotenoids are involved in multiple aspects of plant drought responses, including strengthening antioxidant defenses, regulating root growth, and influencing the composition of root exudate metabolomes.^79^ They are also known for their protective role in photosystems and play a vital role in the response to drought stress.^80^^,^^81^ In the present study, carotenoid contents were significantly higher in plants exposed to TCFs compared to the control. These observations suggest that the T-Consciousness Fields significantly mitigated the detrimental effects of drought stress.

Under drought stress, the soluble protein content typically decreases due to oxidative stress, inhibition of protein synthesis, and metabolic constraints.^82^ Recent experimental data have shown that drought stress significantly reduced the soluble protein content in the leaves of cumin plants.^83^ In contrast, the levels of free amino acids such as proline and other osmolytes increase.^84^ The accumulation of osmolytes contributes to lowering the cellular water potential, facilitating water influx and maintaining turgor pressure.^85^ A study on drought-tolerant transgenic creeping bentgrass (expressing the *ipt* gene to enhance cytokinin synthesis) showed that plants under water deficit maintained higher levels of various stress-responsive proteins compared with non-transgenic controls, thereby contributing to enhanced drought tolerance.^86^ The higher total protein content observed in plants exposed to the TCFs suggests that defense and protective pathways were activated, supporting protein synthesis under stress conditions.

It is well established that one of the most important protective mechanisms in plants under stress is the activation of antioxidant enzymes that scavenge reactive oxygen species.^87^ Among these enzymes, superoxide dismutase (SOD) plays a crucial role as the first line of defense against oxidative stress by catalyzing the conversion of superoxide anions into hydrogen peroxide and molecular oxygen.^88^ By reducing the concentration of superoxide radicals, SOD prevents excessive oxidation and cellular damage, thereby enabling other antioxidant enzymes such as peroxidase and catalase to further decompose hydrogen peroxide.^89^ Numerous studies have shown that treatments which enhance antioxidant defense also increase plant resistance under abiotic stress. For example, exogenous acetate sodium treatment enhanced antioxidant enzyme activity and improved oat tolerance to salinity stress,^90^ while γ-aminobutyric acid (GABA) and melatonin treatments were shown to modulate ROS metabolism and elevate antioxidant defense in pomegranates and cucumbers, respectively.^91^^,^^92^ In the present study, the increased SOD activity indicates an enhanced antioxidant defense system and a reduction in oxidative damage induced by drought stress. This observation is in agreement with our previous study, in which the antioxidant enzyme activities of TCF-treated seedlings were significantly increased under salinity stress.^32^ We suggest expanding this experiment to investigate the potential ameliorative effects of TCFs in improving plant responses to the detrimental impacts of various water regimes, as well as different biotic and abiotic stresses.

Various approaches have been proposed regarding consciousness and mind in plants.^93^ For example, plant neurobiology examines structural similarities, electrical signals, and chemical production in plants compared to animals, suggesting that these similarities indicate the presence of consciousness in plants.^94^ Observations include action potentials in plants that resemble neuronal activity, the accumulation of GABA and glutamate signaling under stress conditions, sensitivity to anesthetic agents, and other phenomena.^95-97^ On the other hand, critics of this approach argue that the prerequisite for the manifestation of consciousness is the evolution of a brain, including the forebrain, midbrain, and hindbrain, along with sensory systems such as vision, smell, touch, and hearing, to enable the creation of an accurate representation of the environment. Therefore, plants, which lack these features, cannot be considered conscious.^98^

The most popular explanations about consciousness have been presented by neuroscience, such as global neuronal workspace,^99^ integrated information theory,^100^ and higher-order thought theory.^101^ Contrary to common views that consider the brain a prerequisite for consciousness,^102^ in Taheri's approach, it is assumed that every particle of existence possesses a level of consciousness and mind, having precise information to determine its own function and essence. Within this framework, different levels of mind are introduced, ranging from the mind of matter and the mind of brainless organisms to the mind of creatures with a brain. To illustrate, this can be likened to a computer, which consists of hardware and software components. The performance and output of the hardware depend entirely on the software, which provides precise instructions and guides the system's operations. Similarly, the physical or “hardware” aspect of every particle of existence requires a corresponding “software” component to define its functions. From this perspective, this “software” part is regarded as the mind. A key challenge in consciousness research is the difficulty of practically and experimentally testing its theoretical models.^103^ In the present study, a hypothesis was examined that proposes the existence of T-Consciousness Fields—fields of a nonenergy and nonfrequency nature whose effects initiate through the human mind.^5^ From this perspective, alongside well-known physical fields such as gravitational and electromagnetic fields, the possibility of fields with nonphysical properties is also considered, which cannot be measured directly with conventional physical instruments. Although direct measurement of these fields is not possible, their existence and interactions with the physical domain can be inferred indirectly through the observation of their effects in the laboratory. This feature motivated us to design experiments with the primary aim of recording the effects of these fields. Two types of TCFs were examined in this study: Faradarmani (T1) and Charge (T2) Consciousness Fields.

It is hypothesized that the application of these fields can induce alterations through the transmission of information. As mentioned earlier, the effects of these fields are initiated through the human mind. Faradarmani is applied without the use of any material substance, whereas in the T2 application, a substance is first exposed to the field and subsequently serves as a medium possessing treatment properties. In the present study, owing to the experimental design and the plant-based system, water was used as the medium. Observing changes in the behavior of samples under the influence of these fields provides preliminary evidence for potential interactions between these fields and plant seedlings.

One of the earliest and most influential models of information theory is the communication model introduced by Shannon and Weaver.^104^ Communication theory assumes that before information is received, a system is characterized by maximum uncertainty, quantified as information entropy, and that this uncertainty may decrease after the information is received.^105^ In an effort to investigate how information transmitted through these fields may influence entropy, a recent EEG-based study examined changes in the entropy of total brain electrical activity under the influence of Faradarmani, and the results demonstrated a significant reduction in entropy.^106^ Similarly, Shannon entropy has been widely used to assess the complexity of neural signals and to quantify the amount of information processed by the brain.^107^

In the Charge Field treatment, water was used as the medium through which the effects of the field were applied. It is therefore possible that the physicochemical properties of the water were altered under the influence of this field, subsequently leading to the observed biological changes; alternatively, the water may simply act as a carrier of information transmitted by the charge field. In the present study, the primary objective was to record biological responses; thus, the physicochemical properties of the water were not analyzed; consequently, any such changes remain unknown. Future studies, in addition to incorporating appropriate sham controls, will focus on comprehensive physicochemical characterization of the treated water, such as measurements of pH, temperature, electrical conductivity (EC), etc. under the influence of TCFs.

## Conclusion

This study demonstrates the potential ameliorative effects of TCFs on wheat seedlings under drought stress. These findings suggest that such treatments may be considered as a qualitative, nonchemical strategies for growers and agricultural stakeholders. Moreover, when the effects of TCFs are observed in brainless entities such as seedlings, the question arises as to how these fields produce such changes. One hypothesis is that transmitted information via these fields can lead to alterations in the characteristics and behavior of the affected sample. Since there is no physical or energetic intervention in this process, another question arises: how does the seedling receive this influence? According to Taheri's perspective, the process of receiving and processing information requires the presence of a level of mind, even in organisms that lack a nervous system. Therefore, the observations in this study may provide empirical evidence supporting these hypotheses. However, further research is needed to gain a deeper understanding of the nature and function of T-Consciousness Fields and the existence of different levels of consciousness and mind in plants.

## Supplementary Material

Supplementary material Supplementary Material.

## Data Availability

Harvard Dataverse: Influence of T-Consciousness Fields on Germination, Growth, and Biochemical Responses of Wheat under Drought Stress, https://doi.org/10.7910/DVN/IBT9WM.
